# Clinicopathologic features, treatment, and prognosis of pregnancy-associated breast cancer

**DOI:** 10.3389/fonc.2022.978671

**Published:** 2022-12-14

**Authors:** Yuechong Li, Yingjiao Wang, Qiang Sun, Songjie Shen

**Affiliations:** Department of Breast Surgery, Peking Union Medical College Hospital, Peking Union Medical College, Chinese Academy of Medical Sciences, Beijing, China

**Keywords:** pregnancy-associated breast cancer, prognosis, clinical features, treatment, event-free survival

## Abstract

**Purpose:**

To identify the clinicopathological features, treatment, and prognosis of patients with breast cancer, who were diagnosed during and after pregnancy.

**Methods:**

We searched for patients with pregnancy-associated breast cancer (PABC) using the big data query and analysis system of Peking Union Medical College Hospital from between January 1, 2013, and December 31, 2021, and matched each patient with two non-PABC patients by age at diagnosis, year at diagnosis, and tumor stage. The clinicopathologic features, treatment, and outcomes of breast cancer during pregnancy (BC-P) and breast cancer during the first-year post-partum (BC-PP) were examined retrospectively in two case-control studies.

**Results:**

Eighteen BC-P cases, 36 controls for BC-P cases, 62 BC-PP cases, and 124 controls for BC-PP cases were enrolled in our study. The expression of HER-2 and Ki-67 was higher in BC-PP cases than in its controls (*P=*0.01, 0.018, respectively). Patients with BC-PP were more likely to choose mastectomy than breast-conserving surgery (*P*=0.001). There were no significant differences in event-free survival (EFS) between patients with BC-P and BC-PP and their controls.

**Conclusion:**

BC-P and BC-PP patients displayed adverse clinicopathological features in our population. However, when matched by age at diagnosis, year of diagnosis, and tumor stage, BC-P and BC-PP patients did not show inferior outcomes to controls, probably due to aggressive multimodality therapy.

## Introduction

Pregnancy-associated breast cancer (PABC) is generally defined as breast cancer diagnosed during pregnancy or within 1 year of delivery (1–3). The incidence of PABC ranges from 1 in 10,000 to 1 in 3000 pregnancies, representing only 0.2–3.8% of overall breast cancer cases (1), and it is one of the most commonly diagnosed cancers during pregnancy and lactation (4–6). The peak age of breast cancer in Chinese women is approximately 45 years, 10 to 20 years earlier than that in women in Europe and the United States (7). Further, the average childbearing age continues to rise due to abolishment of the restriction that a couple could have only one child in 2016 and subsequently decreased restrictions to allow a third child in 2021. Meanwhile, the introduction of non-invasive prenatal testing (NIPT) that has increased cancer detection in asymptomatic pregnant patients in the developed countries, leading to early diagnosis (8–10). Therefore, it can be predicted that the incidence of PABC in China will increase in the future. With a deeper understanding of the biology and clinical features of PABC, researchers tend to divide this entity into two major groups: breast cancer diagnosed during pregnancy (BC-P) and breast cancer diagnosed post-partum (BC-PP) (11). Increasing evidence suggests that these two entities likely differ in biology and prognosis (3, 11).

However, existing literature provides a mixed view of whether PABC confers a worse prognosis than non-PABC. Few studies on the prognosis of PABC have been conducted in China. Thus, we need a thorough understanding of the clinicopathological features, treatments, and prognosis of patients with PABC. We performed a retrospective case-control study at Peking Union Medical College Hospital (PUMCH), aiming to provide a reference for the diagnosis and treatment of PABC.

## Methods

### Patient selection

**Figure 1:**
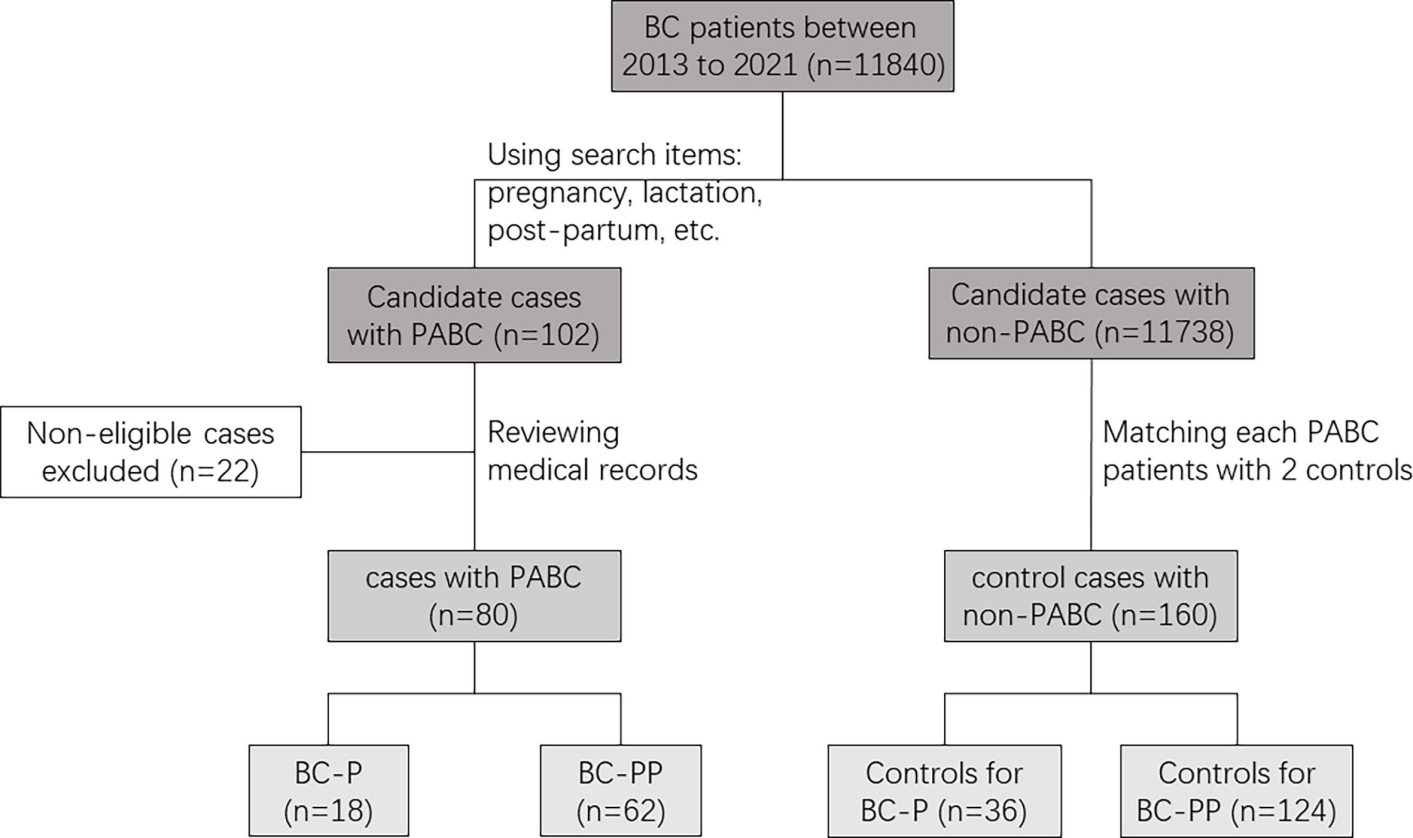
Diagram of study population.

Using the big data query and analysis system of PUMCH, we collected 264 records from between January 1, 2013, and December 31, 2021, using the following search terms: pregnancy, pregnant, post-partum, lactation, gestation, delivery, breastfeeding, and breast cancer (**Figure 1**). After reviewing the medical records of patients suspected of having PABC to ensure that breast cancer (BC) was diagnosed during pregnancy or within a year after delivery, 80 patients with PABC were enrolled (18 were diagnosed during pregnancy, and 62 were diagnosed within 1 year after delivery). We created two separate case groups (BC-P and BC-PP) and two separate corresponding control groups. BC-P patients were diagnosed during pregnancy, and BC-PP patients were diagnosed during the first year of delivery. For each patient, we matched two patients with breast cancer who were not diagnosed during pregnancy or lactation according to age ( ± 2 years), year of diagnosis ( ± 2 years), and TNM stage.

### Data collection

Data regarding age at diagnosis, family history, pathological characteristics (pathological type, TNM stage, tumor size, nodal involvement, NPI index, histological grade, estrogen receptor (ER) and progesterone receptor (PR) status, expression of human epidermal growth factor receptor-2 (HER-2), expression of Ki-67, etc.), treatment (type of surgery, chemotherapy, radiotherapy, endocrine therapy, and target therapy), date of relapse, and site of relapse were obtained by chart review. TNM stage was evaluated according to the 8th guideline of the American Joint Committee on Cancer (AJCC) (12). The Nottingham Prognostic Index (NPI) was calculated using the following equation: NPI = 0.2 × tumor size (cm) + grade (1–3) + lymph node status (1–3) (13). NPI grade was determined according to NPI: Grade 1 if the NPI was less than 3.4, Grade 2 if the NPI was between 3.4 and 5.4 and Grade 3 if the NPI was over 5.4. ER, PR, HER-2 and Ki-67 expressions were evaluated by immunohistochemistry (IHC), as previously described (14). Fluorescence *in situ* hybridization (FISH) was performed for tumors with a HER-2 score of +2 by IHC. Tumors were divided into four breast cancer subtypes, luminal-A, luminal-B, HER-2 positive and triple-negative, using the expression of ER, PR, HER-2 and Ki-67 (15).

### Statistical analysis

Pearson chi-square or Fisher’s exact tests were used to compare the pathological characteristics and treatment of patients among different subgroups, as appropriate. The survival endpoints were event-free survival (EFS) and overall survival (OS). EFS was defined as the time from diagnosis to any locoregional or distant recurrence, contralateral breast cancer, disease progression, or death from any cause, whichever occurred first. OS was defined as the time from diagnosis to death due to any cause. The Kaplan–Meier method was used to perform survival analysis. Cox proportional hazards models were used to estimate the hazard ratios (HR) with 95% confidence intervals (CI) for recurrence for each case group compared to its control group. Univariate and multivariate analyses were used to evaluate other factors associated with recurrence. All tests were two-sided, and a P-value< 0.05 was considered statistically significant. All statistical analyses were performed using the SPSS statistical software (version 25.0; IBM Corporation, Armonk, NY, USA).

## Results

### Study population and clinicopathological features

Eighty patients (18 with BC-P and 62 with BC-PP) and 160 controls were enrolled (**Table 1**). The percentage of PABC among diagnosed breast cancers between 2013 and 2021 in PUMCH was 0.68%. The median age of patients with PABC was 38.5 years old (range, 25–48). More than 60% of PABC cases were stage II or higher. After matching, 16 BC-PP patients (25.8%) had a family history of breast cancer, compared to seven cases (5.6%) in the BC-PP controls (*P* < 0.001). There were no significant differences in the distribution of lymph node invasion, histological grade, NPI grade, histological type, ER/PR status, or molecular subtypes between each case and control groups. However, when comparing BC-PP cases and controls, the expression of HER-2 and Ki-67 was higher in BC-PP cases (*P*=0.01, 0.018, respectively). Among the five stage IV patients with PABC, four were of the luminal B type and one was triple-negative (**Supplementary Table 1**). All stage IV PABC patients underwent surgical treatment, postoperative radiotherapy, and chemotherapy, among which one patient received only palliative bilateral mastectomy. Three patients developed disease progression after surgery, among which one patient developed local progression, one had local progression and death, and one had progression of bone metastases.

**Table 1:** Clinicopathological features and treatment of PABC patients.

Characteristic	BC-PP (n = 62)	BC-PP controls (n = 124)	*P* value	BC-P (n = 18)	BC-P controls (n = 36)	*P* value
Age groups (years old)			0.117			1
≤35	38(61.3)	90(72.6)		10(55.6)	20(55.6)	
>35	24(38.7)	34(27.4)		8(44.4)	16(44.4)	
Family history			<0.001			0.107
Yes	16(25.8)	7(5.6)		6(33.3)	4(11.1)	
No	46(74.2)	117(94.4)		12(66.7)	32(88.9)	
Tumor size(cm)	2.9	2.5	0.134	3.4	2.5	0.358
Node invasion			0.427			0.578
Negative	29(46.8)	73(59.3)		7(38.9)	12(33.3)	
Positive	33(53.2)	50(40.7)		10(55.6)	24(66.7)	
Histological grade			0.468			0.700
G1/G2	28(45.2)	63(50.8)		8(44.4)	18(50.0)	
G3	34(54.8)	61(49.2)		10(55.6)	18(50.0)	
TNM stage			1			1
0	7(11.3)	14(11.3)		0	0	
I	12(19.4)	24(19.4)		3(16.7)	6(16.7)	
II	22(35.5)	44(35.5)		8(44.4)	16(44.4)	
III	17(27.4)	34(27.4)		6(33.3)	12(33.3)	
IV	4(6.5)	8(6.5)		1(5.6)	2(5.6)	
Histological type			0.738			—
Invasive	55(88.7)	107(87.0)		18(100)	36(100.0)	
DCIS	7(11.3)	16(13.0)		0	0	
NPI grade			0.533			0.18
1	26(41.9)	46(37.4)		6(33.3)	12(33.3)	
2	24(27.5)	58(47.2)		9(50.0)	12(33.3)	
3	12(19.4)	19(15.4)		2(11.1)	12(33.3)	
ER status			0.173			0.298
Positive	38(63.3)	90(73.2)		12(66.7)	30(83.3)	
Negative	22(36.7)	33(26.8)		6(33.3)	6(16.7)	
PR status			0.098			0.425
Positive	34(56.7)	85(69.1)		10(55.6)	24(66.7)	
Negative	26(43.3)	38(30.9)		8(44.4)	12(33.3)	
HER-2 expression			0.010			0.101
Positive	28(50.9)	35(30.4)		9(50.0)	10(27.8)	
Negative	27(49.1)	80(69.6)		8(44.4)	24(66.7)	
Ki-67 expression			0.018			1
<20%	6(10.2)	31(25.2)		3(16.7)	7(19.4)	
≥20%	53(89.8)	92(74.8)		15(83.3)	29(80.6)	
Tumor subtype			0.069			—
Luminal A	2(3.2)	19(17.8)		2(11.1)	4(11.1)	
Luminal B	33(60.0)	60(56.1)		10(55.6)	26(72.2)	
HER-2 positive	9(16.4)	14(13.1)		3(16.7)	3(8.3)	
Triple-negative	11(20.0)	14(13.1)		3(16.7)	3(8.3)	
Neoadjuvant chemotherapy			0.186			0.107
Yes	7(11.3)	6(4.8)		6(33.3)	4(11.1)	
No	55(88.7)	118(95.2)		12(66.7)	32(88.9)	
Surgery type			0.001			0.083
Mastectomy	53(85.5)	78(62.9)		17(94.4)	25(69.4)	
Breast-conserving	9(14.5)	46(37.1)		1(5.6)	11(30.6)	
chemotherapy			0.899			0.186
Yes	47(82.5)	98(81.7)		12(66.7)	35(97.2)	
No	10(17.5)	22(21.7)		2(11.1)	1(2.8)	
Radiotherapy			0.454			0.529
Yes	39(69.6)	90(75.0)		8(44.4)	24(66.7)	
No	17(30.4)	30(25.0)		6(33.3)	12(33.3)	
Endocrine therapy			0.092			0.581
Yes	38(61.3)	91(73.4)		10(55.6)	30(83.3)	
No	24(38.7)	33(26.6)		4(22.2)	6(16.7)	
Target therapy			0.021			0.052
Yes	22(39.3)	27(22.5)		8(57.1)	10(27.8)	
No	34(60.7)	93(77.5)		6(42.9)	26(72.2)	

The bold values indicate a P value less than 0.05.

### Treatment

We found no significant differences in the proportions of chemotherapy, radiation, and endocrine therapy between the case and control groups. BC-PP patients had a higher rate of target therapy than controls (*P*=0.021). Interestingly, BC-PP patients were more likely to choose mastectomy, rather than breast-conserving surgery, than their controls (*P*=0.001). Among the 18 BC-P patients, three chose to terminate their pregnancy because they were still in the first trimester of pregnancy (less than 7 weeks). Seven patients underwent surgery during pregnancy, all of whom were in the second or third trimester of pregnancy. Five patients received neoadjuvant therapy during pregnancy, followed by surgery after delivery, and three patients did not receive treatment during pregnancy, followed by corresponding treatment after delivery.

### Survival

**Figure 2:**
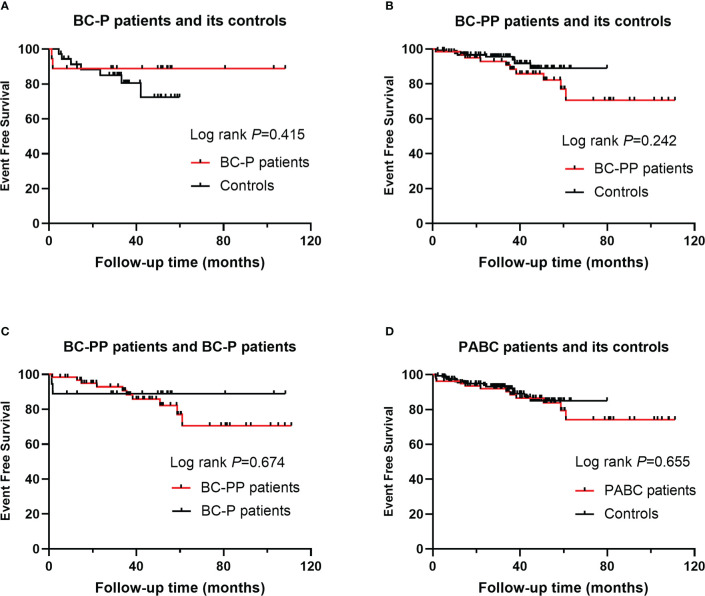
Event free survival (EFS) for PABC patients and their controls: **(A)** EFS for BC-P patients and its controls; **(B)** EFS for BC-PP patients and its controls; **(C)** EFS for BC-P patients and BC-PP patients; **(D)** EFS for PABC patients and its controls.

For BC-P cases and its controls, the median follow-up time was 35.1 months (range, 1.2 months to 108.3 months). The 5-year EFS of the BC-P patients was 88.9%, and the EFS of the control patients was 72.5% (*P*=0.415) (**Figure 2A**). Among the BC-PP cases and controls, the median follow-up time was 35.1 months (range, 1.4 months to 111 months). The 5-year EFS of BC-PP patients was 80.4%, while that of the control patients was 89.0% (*P*=0.242) (**Figure 2B**). There was no significant difference between the EFS of BC-P and BC-PP patients (*P*=0.674) or between the EFS of PABC patients and its controls (*P*=0.655). (**Figures 2C, D**). In the univariate analysis, the hazard of recurrence was twice as high for pathological grade 3 patients than for grade 1 and 2 patients, the difference of which was significant (HR 2.398, 95% CI 1.033–5.567, *P*=0.042). No significant differences were found in other factors among PABC patients or other candidates in univariate and multivariate analyses (**Tables 2**, **3**). We also calculated the EFS of PABC patients after excluding stage IV patients and found no significant differences between the different comparison groups (**Supplementary Figure 1**). Due to the limited number of deaths (only two cases, one in the BC-PP group and the other in the control of the BC-PP group), we did not calculate overall survival. In the subgroup analysis, we found no significant differences among the subgroups (**Table 4**).

**Table 2:** Factors associated with recurrence among PABC patients and their controls.

	Univariate			Multivariate		
	HR^a^	95% CI^b^	*P* value	HR	95% CI	*P* value
Case vs control	1.194	0.547-2.607	0.656	1.015	0.399-2.580	0.975
Age	1.003	0.928-1.085	0.935	1.004	0.919-1.096	0.933
Family history (Yes vs No)	0.538	0.159-1.825	0.320	0.425	0.090-2.000	0.279
Surgery type (breast-conserving vs mastectomy)	0.799	0.321-1.989	0.630	0.991	0.360-2.727	0.987
Ki-67 (≥20% vs<20%)	2.020	0.603-6.759	0.254	1.822	0.373-8.894	0.459
Pathological grade (G3 vs G1/2)	2.398	1.033-5.567	0.042	1.999	0.758-5.267	0.161
ER (positive vs negative)	0.578	0.260-1.280	0.180	1.249	0.264-5.904	0.779
PR (positive vs negative)	0.488	0.222-1.069	0.073	0.681	0.147-3.153	0.623
HER-2 (positive vs negative)	1.499	0.683-3.292	0.313	1.549	0.657-3.653	0.317

a HR hazard ratio.

b CI confidence interval.

**Table 3:** Factors associated with recurrence among BC-P patients and BC-PP patients.

	Univariate			Multivariate		
	HR^a^	95% CI^b^	*P* value	HR	95% CI	*P* value
BC-P versus BC-PP	0.723	0.158-3.307	0.676	1.199	0.186-7.747	0.849
Age	1.002	0.893-1.124	0.977	1.017	0.881-1.175	0.814
Family history (Yes vs No)	0.584	0.153-2.225	0.431	0.475	0.088-2.558	0.386
Surgery type (breast-conserving vs mastectomy)	1.463	0.316-6.780	0.627	2.005	0.356-11.285	0.430
Pathological grade (G3 vs G1/2)	2.426	0.642-9.170	0.192	1.869	0.451-7.746	0.388
ER (positive vs negative)	0.406	0.123-1.338	0.139	0.543	0.140-2.111	0.378
HER-2 (positive vs negative)	2.125	0.638-7.071	0.219	4.392	0.909-21.220	0.066

a HR hazard ratio.

b CI confidence interval.

**Table 4:** Subgroup analysis of PABC patients and their controls.

Subgroup	PABC		non-PABC		HR^a^ (95% CI^b^)	*P* value
	No. of Patients	Events	No. of Patients	Events		
**Family history**
yes	22	3	11	0	–	–
no	58	9	149	15	1.258 (0.544-2.909)	0.591
**Ki-67**
≥20%	68	11	121	11	1.269 (0.535-3.006)	0.589
<20%	9	0	38	3	–	–
**Her-2 status**
positive	37	8	45	4	1.890 (0.556-6.426)	0.308
negative	35	4	104	9	1.010 (0.307-3.324)	0.987
**Tumor subtype**
luminal A	4	0	23	1	–	–
luminal B	43	6	86	9	0.910 (0.308-2.688)	0.865
Her-2 positive	12	4	17	3	1.917 (0.421-8.719)	0.4
TNBC	14	2	17	1	2.131 (0.191-23.785)	0.539
**Surgery type**
mastectomy	70	10	103	13	0.985 (0.422-2.299)	0.971
breast-conserving	10	2	57	2	2.891 (0.393-21.235)	0.297
**Target therapy**
yes	30	6	37	3	2.101 (0.509-8.671)	0.305
no	40	6	119	12	1.041 (0.377-2.875)	0.938

a HR hazard ratio.

b CI confidence interval.

## Discussion

In our study, we found that more BC-PP patients had a family history of breast cancer (25.8%) compared to the control group. Previous studies have confirmed that family history is a major risk factor for breast cancer (16), as it was found that nearly a quarter of all breast cancer cases are related to a family history (17). Recently, an accumulating amount of research observed multiple differentially expressed genes and numerous non-silent mutations in PABC patients (18). Through microarray assay, Nguyen et al. (19)demonstrated that PABC group contained a significantly higher number of non-silent mutations than non-PABC group. They found that TP53 and PIK3CA were the most frequently mutated genes. Azim et al. (20)draw the same conclusion, and they also found that the expression of two particular pathways was significantly enriched in PABC group: the G-protein coupled receptor pathway (GPCR) and the serotonin receptor signaling pathway. Zografos et al. (21)observed that among a cohort of 20 PABC patients, 35% of PABC patients tested carried pathogenic mutations in BRCA1 and CHEK2 genes. Some patients carrying germline mutations did not report a family history of cancer (21).Thus, highlighting the genomics of PABC and examining all biological pathways will further facilitate the identification of novel biomarkers defining women who are at high risk of developing PABC in the general population (18, 22). BC-PP is also more unfavorable, as the rate of HER-2 positive and Ki-67≥20% was significantly higher than that of its controls (P=0.01, 0.018, respectively), although age, year of diagnosis, and TNM stage were well matched. Her-2 positive and high Ki-67 expression are two major IHC markers associated with poorer prognosis, especially before Her-2 target therapy. Therefore, we anticipate that BC-PP has adverse clinicopathological features that may affect the prognosis. However, we did not find a significant difference in the percentage of ER/PR-positive tumors in PABC patients compared to their controls, although there seemed to be a tendency for more ER/PR-negative tumors. Most studies have found that PABC is associated with a high frequency of ER/PR-negative tumors. A large population-based cohort showed that more than 50% of PABC cases are ER/PR-negative, with a significant difference compared with non-PABC cases (23). A recent prospective study also found that patients with PABC had lower hormone receptor expression and higher levels of HER2 overexpression (24). However, these two studies did not match tumor stage of both groups.

Overall, the treatment for PABC differs little from standard therapy for breast cancer, but the timing of treatment during pregnancy is important. According to the latest National Comprehensive Cancer Network clinical practice guideline for breast cancer, surgery is the preferred treatment for PABC. If the tumor is unresectable, neoadjuvant chemotherapy is usually initiated in the middle and late stages of pregnancy. Radiotherapy, endocrine therapy, and targeted therapy are not permitted during pregnancy. In this study, all the patients underwent surgical treatment. It is worth mentioning that the proportion of PABC patients who chose mastectomy was higher, which is consistent with the findings of Johansson et al. (25). The choice of breast conserving surgery for patients with PABC remains controversial. Breast cancer patients undergoing breast-conserving surgery have a higher quality of life than those receiving mastectomy (26). A multicenter study has proven that the long-term survival of patients undergoing breast-conserving surgery and following radiotherapy is not different from that of patients undergoing mastectomy (27). However, in-breast metastasis is common in patients with PABC. Researchers thought that this was the result of the promotional effects of pregnancy-associated hormones and delay in diagnosis. Further studies have revealed that involution, in which the mammary gland regresses to its pre-pregnancy state, may be a crucial point. During this process, the microenvironment of the involuting mammary glands have attributes of inflammation and wound healing, which can promote tumorigenesis (28). Cancer cells migrate more easily along dilated milk-producing lobules, simultaneously increasing the risk of local regional recurrence (29). A case-control study showed that radical surgery is the preferred first-line treatment for patients with PABC (30). This may be due to patients’ greater fear of breast cancer and lower desire to maintain breast appearance during pregnancy and lactation. Overall, the treatment options during pregnancy are influenced by many factors. A supportive multidisciplinary team are warranted including neonatologists, perinatologists, oncologists, obstetricians, teratologists, toxicologists and psychologists during the pregnancy and after birth.

Age and stage are two important factors that affect breast cancer prognosis. During pregnancy or lactation, the mammary gland swells, making it difficult to detect early lesions, resulting in a higher tumor stage at diagnosis. To eliminate the impact of other features on the prognosis of PABC, we strictly matched patients with PABC with two controls by age, stage, and year at diagnosis. However, we did not confirm that PABC was associated with significantly inferior outcomes compared with non-PABC controls. A recent meta-analysis also found that the negative effect on OS and DFS appeared to be less pronounced than in previous studies (31). In addition, HER-2 positive tumors had the worst prognosis of subtypes before the introduction of HER-2 target therapy (32). After Trastuzumab was covered by the National Health Insurance in 2017, the prognosis of this subtype greatly improved, which may partly explain the small difference between the outcomes of PABC and non-PABC. All patients in our study underwent surgery, and over 80% received chemotherapy, demonstrating the benefit of aggressive multi-modal treatment in overcoming PABC’s adverse biological features. Over the years, researchers have found that the prognosis of PABC is closely related to the definition of PABC. Definitions of the duration of the post-partum period have been controversial, and this variability may lead to diverse results regarding the prognosis. A meta-analysis of 76 studies suggested that the definition of PABC should be extended to include patients diagnosed up to approximately 6 years post-partum (31). For PABC diagnosed one year after the last delivery, the mortality rate was almost 60% higher. At the same time, another cohort study found that the prognosis of patients with PABC was different at different stages of pregnancy and lactation (33). An accurate definition of PABC is important. To better understand the biological mechanism and pathophysiological process underlying PABC, better classification is required to guide the clinical diagnosis and treatment of PABC.

The limitations of our study include its retrospective, single-institution design and relatively small sample size. Nevertheless, we described the clinicopathological features, treatment, and prognosis of both BC-P and BC-PP. By matching age, tumor stage, and year of diagnosis, we minimized the effects of treatment schedule progress, age, and stage on prognosis as much as possible, making the results more reliable.

In conclusion, after matching for age, tumor stage, and year at diagnosis, PABC still has some adverse characteristics. PABC patients’ prognosis was not significantly worse than that of non-PABC patients, a finding that we believe is due to the aggressive multimodality treatment and well-balanced age distribution and tumor stage in the study. To study outcomes and ensure optimal treatment delivery for women with PABC, larger multi-institutional studies are needed in the future. Further study of the pregnant and post-partum breast microenvironment is urgently needed to elucidate tumorigenesis and metastasis of PABC, facilitating the development of prevention and treatment agents.

## Data availability statement

The raw data supporting the conclusions of this article will be made available by the authors, without undue reservation.

## Ethics statement

The studies involving human participants were reviewed and approved by Ethics Review Committee of Peking Union Medical College Hospital, Chinese Academy of Medical Sciences. Written informed consent for participation was not required for this study in accordance with the national legislation and the institutional requirements.

## Author contributions

All authors contributed to the study conception and design. Material preparation, data collection and analysis were performed by YL, YW, and SS. The first draft of the manuscript was written by YL and SS, and all authors commented on previous versions of the manuscript. All authors read and approved the final manuscript.
