# Supplementary material for: Clinicopathologic features, treatment, and prognosis of pregnancy-associated breast cancer
Source: Front Oncol. 2022 Dec 14;12:978671. doi: 10.3389/fonc.2022.978671 (PMC9795172; doi:10.3389/fonc.2022.978671)
Supplement: Supplementary file 1 [file DataSheet_1.docx]

Supplementary Material


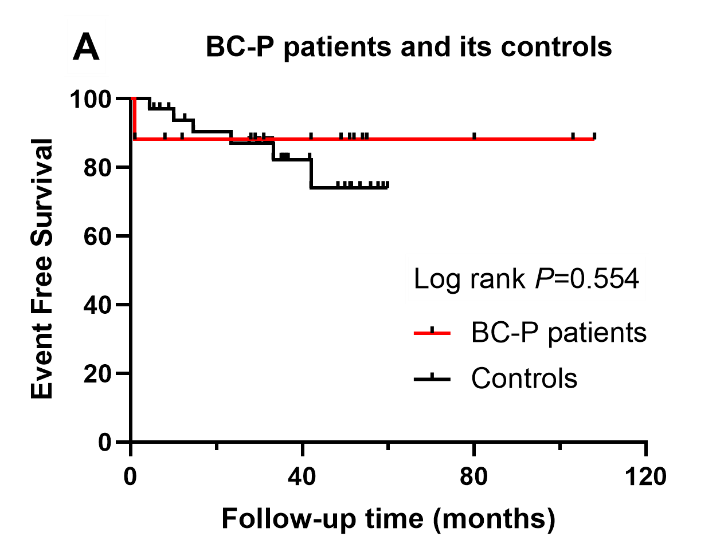

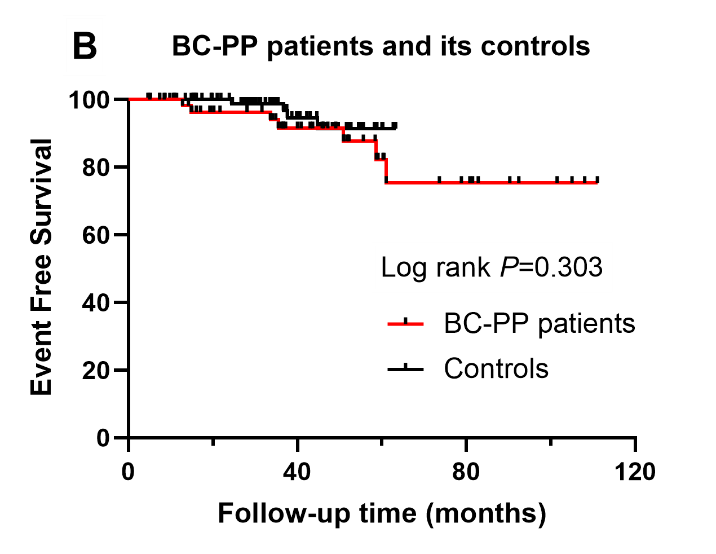


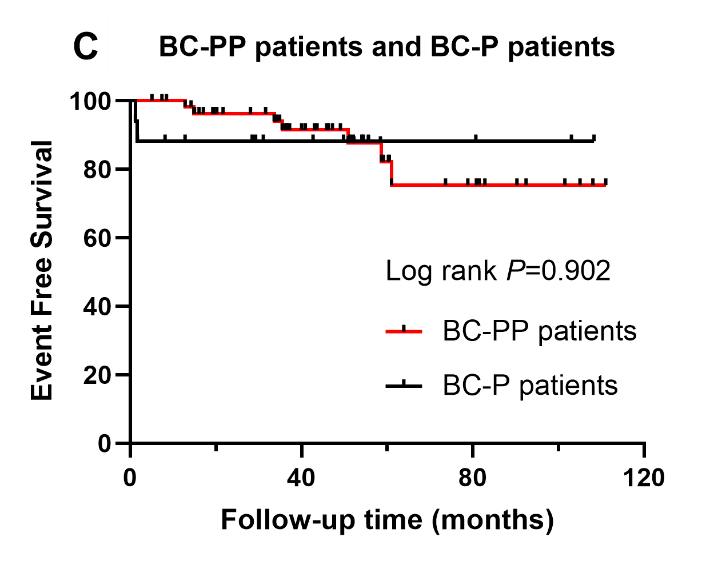

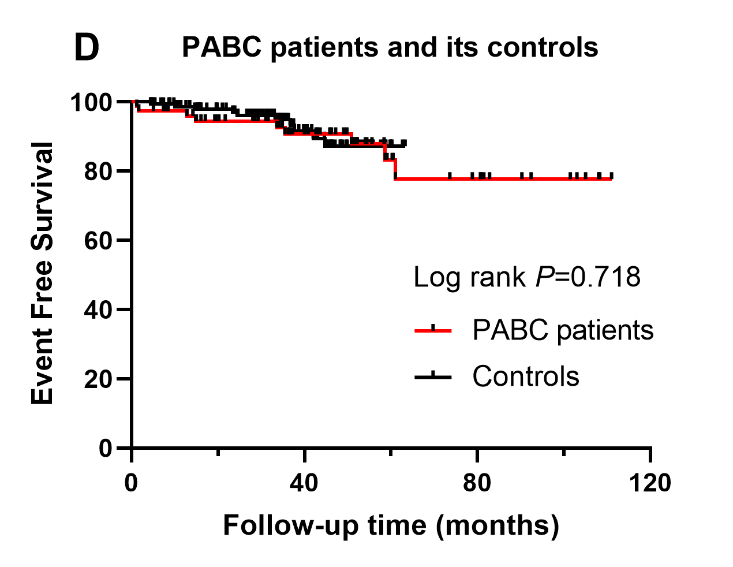


**Figure S1.** Event free survival (EFS) for PABC patients without stage IV and their controls. **(A)** EFS for BC-P patients and its controls, **(B)** EFS for BC-PP patients and its controls, **(C)** EFS for BC-PP patients and BC-P patients, **(D)** EFS for PABC patients and its controls

**Table S1** Characteristics of PABC patients with stage IV tumors

| Characteristics | PABC（n=5） | controls（n=10） |
| --- | --- | --- |
| Tumor subtype |  |  |
| Luminal A | 0 | 1 |
| Luminal B | 4 | 7 |
| HER-2 positive | 0 | 2 |
| Triple-negative | 1 | 0 |
| Treatment |  |  |
| Neoadjuvant chemotherapy | 4 | 4 |
| Mastectomy | 4 | 10 |
| Chemotherapy | 5 | 9 |
| Radiotherapy | 5 | 8 |
| Endocrine therapy | 4 | 8 |
| Target therapy | 2 | 3 |
| Metastasis site |  |  |
| Cervical lymph node | 2 | 1 |
| Bone | 2 | 8 |
| Lung | 1 | 1 |
| Liver | 2 | 2 |
| Endometrium | 0 | 1 |
| Relapse | 3 | 5 |
